# A Prospective Study to Evaluate the Role of Combined Diagnostic Laparoscopy and Hysteroscopy in the Management of Female Infertility

**DOI:** 10.7759/cureus.52170

**Published:** 2024-01-12

**Authors:** Parul Sharma, Ambika Jhanwar, Kamlesh Kumari, Jyoti Arya, Bhavna Bharti, Bushra Majeed, Daxita Dabas

**Affiliations:** 1 Department of Obstetrics and Gynaecology, Jaipur National University, Institute for Medical Sciences and Research Centre, Jaipur, IND

**Keywords:** hysterosalpingography, infertility, chromopertubation, laparoscopy, hysteroscopy

## Abstract

Introduction

Infertility affects approximately 10-15% of couples worldwide. Hysteroscopy and laparoscopy are two newer modalities available for the evaluation of infertility and are complementary rather than mutually exclusive. Each provides useful information that the other may not have and each has its advantages.

Materials and methods

A total of 75 patients of female infertility (study group) in the age group of 18-40 years from the Outpatient Department (OPD) were recruited. Infertility was defined as one year of unprotected intercourse without pregnancy. Hysteroscopy and laparoscopy were carried out in each patient at the follicular phase of the menstrual cycle. Hysteroscopic findings were compared with laparoscopic findings for uterine and tubal pathology. Hysteroscopy as a procedure was also compared with laparoscopy as a one-step procedure for diagnostic accuracy in investigating a case of female infertility. The data was analyzed by Statistical Package for the Social Sciences (IBM SPSS Statistics for Windows, IBM Corp., Version 29.0, Armonk, NY).

Observations and results

In our study, out of a total of 75 cases evaluated for infertility, primary infertility patients were 48 (64%) and secondary infertility patients were 27 (36%). In our study, both tubes were patent on chromopertubation in primary infertility (PI) vs secondary infertility (SI) in 49.33% vs 21.33% of total cases. Both tubes were blocked in PI vs SI in 9.33% vs 8% of total cases. In our study, 20 patients (26.66%) underwent hysteroscopic intervention. Adhesiolysis was the commonest procedure required in seven (9.33%) followed by hysteroscopic cannulation in six (8%). In our study, a total of 30 procedures were performed in 20 patients during laparoscopy. The most common procedure required was ovarian drilling in 22.66% (17/75) followed by surgery for endometriosis in 10.66% (8/75). Adhesiolysis was required in 5/75 (6.66%). Both laparoscopy and hysteroscopy were normal in 44/75 cases for uterine findings.

Conclusion

Thus, hysterolaparoscopy as “one step” had various advantages in our study, more accuracy in the findings and therapeutic intervention in the same sitting reducing the cost. The addition of hysteroscopy to laparoscopy is invaluable in the infertility workup as it has a definite edge in the detection of uterine pathology, as well as being therapeutic at the same time. More accuracy in the diagnostic findings and therapeutic intervention in the same setting will help in reducing the time and cost of treatment.

## Introduction

Infertility affects approximately 10-15% of couples worldwide [1]. Overall incidence of infertility has remained relatively unchanged over the past three decades. However, the evaluation and treatment of infertility have changed remarkably during these years [2]. Tuboperitoneal pathology is responsible for infertility in 40-50% of the cases while uterine pathology accounts for 15-20% of cases [3,4]. Other factors include ovulatory dysfunction (30-40%) and male factors (30-40%) [5]. Unexplained infertility accounts for 10% of cases and is the diagnosis of exclusion given to couples who have completed a standard infertility evaluation with no abnormal findings [6].

Traditional ways to assess the uterine cavity, tubal structure, and tubal patency was hysterosalpingography (HSG) but it has now been largely superseded by hysteroscopy and laparoscopy [7]. In this scenario, the role and place for newer and high-tech methods like laparoscopy and hysteroscopy needs to be adequately established, so that it is neither overused nor the patients who can benefit from it are deprived of it. Hysteroscopy and laparoscopy are two newer modalities available for the evaluation of infertility and are complementary rather than mutually exclusive. Each provides useful information that the other may not have and each has its advantages [8]. Since laparoscopy is an important procedure, combining it with hysteroscopy at the same sitting ("one step" procedure) may even obviate the need for HSG for "in milieu" pathology in the uterus. With this in mind, the benefits of a procedure like hysterolaparoscopy were studied in our study. All women were subjected to hysterolaparoscopy at the same sitting in the follicular phase of the cycle.

## Materials and methods

This study is an observational prospective study of female infertility patients, conducted from October 2021 to September 2023. The study was carried out in the Department of Gynaecology and Obstetrics at Jaipur National University (JNU) Hospital, Jaipur, India which is an important tertiary care referral centre in western India. Approval for the study was obtained from the Institutional Ethics Committee, letter no. IEC No. JNUIMSRC/IEC/2021/36.

A total of 75 patients of female infertility (study group) in the age group of 18-40 years from the Outpatient Department (OPD) were recruited. Inclusion and exclusion criteria are listed in Table 1. Infertility was defined as one year of unprotected intercourse without pregnancy. This is further classified as primary infertility, in which no previous pregnancies have occurred, and secondary infertility, in which a prior pregnancy, although not necessarily a live birth has occurred.

**Table 1:** Inclusion and exclusion criteria

Inclusion criteria	Exclusion criteria
Female infertility patients (primary or secondary infertility)	Age <18 years or >40 years
Age group of 18 to 40 years	Active genital infection
	Contraindication for either hysteroscopy or laparoscopy
	Lower genital tract malignancies
	Male factor infertility

Women with active genital infections or having contraindications for either hysteroscopy or laparoscopy were excluded.

Detailed history and general physical and gynecological examination were done and recorded in a pre-designed proforma. Basic tests like husband’s semen analysis, hormonal assays, ultrasonography, and premenstrual endometrial biopsy were carried out and the study group was selected concerning appropriate inclusion and exclusion criteria.

Hysteroscopy and laparoscopy were carried out in each patient. In secondary infertility cases, past obstetric history, mode of delivery, and other parameters were collected using a structured proforma.

The sample size was based on clinical experience and a review of the literature; it was estimated that n= 75 would be statistically significant.

Hysterolaparoscopy

The procedure was carried out in the follicular phase of the menstrual cycle (days 7-8). This is done to avoid retrograde menstruation. This timing also helps by increasing the chances of fertilization by its therapeutic potential. It was done as an in-patient procedure under general anesthesia. Storz laparoscope (10 mm diameter) (Karl Storz, Tuttlingen, Germany) was introduced after creating pneumoperitoneum infra umbilically. During the procedure, a thorough inspection of the uterus (surface and shape of the uterus), anterior and posterior cul-de-sacs, fallopian tubes, ovaries, ovarian fossae, and rest of the pelvic peritoneum was performed and any abnormality was noted down including any adhesions. Chromopertubation (CPT) was performed in all the cases.

**Figure 1:**
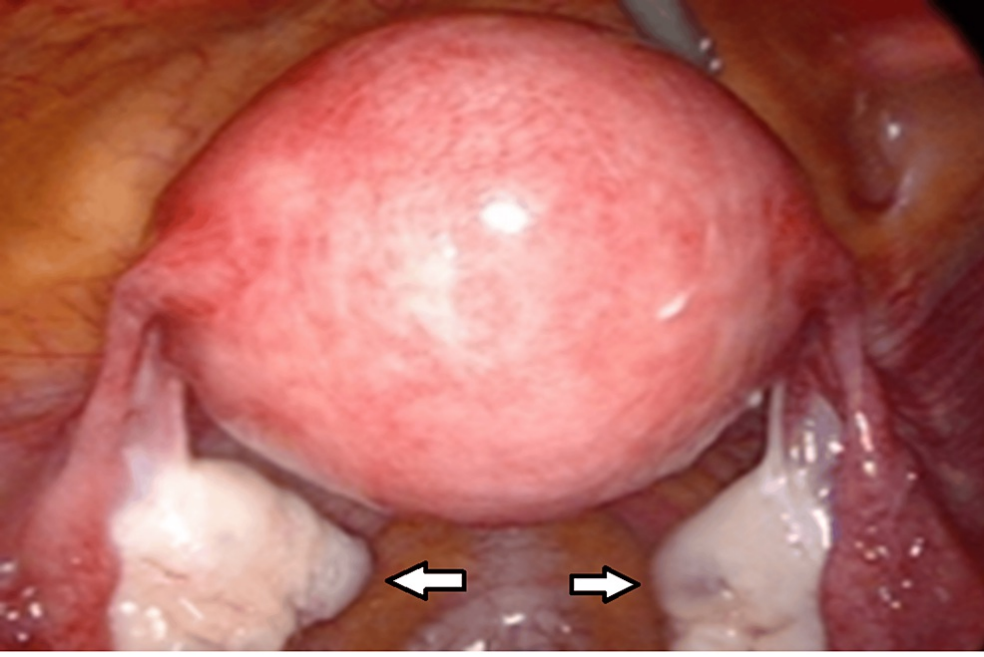
Normal laparoscopic view Arrows indicating normal bilateral ovaries and fallopian tubes.

Normal laparoscopy was labeled in the absence of any pathology (Figure 1).

**Figure 2:**
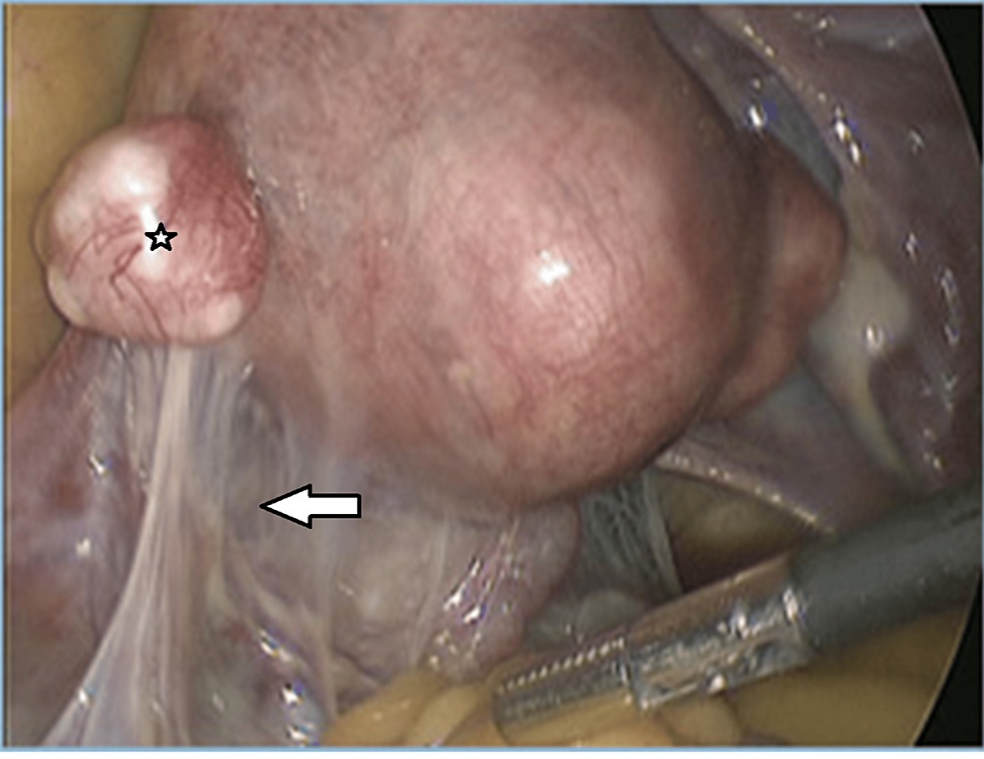
Laparoscopic view of pelvic adhesions The arrow indicating fibrous adhesions and a star showing a subserosal fibroid.

Abnormal laparoscopic findings were labeled in the presence of tubal occlusion (unilateral or bilateral), adhesions - peritubal, peritoneal, perihepatic, endometriosis, small fibroid, paratubal or fimbrial cysts, etc. (Figure 2).

Hysteroscopy

Storz hysteroscope (5 mm diameter) (Karl Storz, Tuttlingen, Germany) was used for diagnostic hysteroscopy. After cleaning the vagina and cervix with antiseptic lotion, the cervix was held with vulsellum. Storz hysteroscope was introduced into the uterus via the cervix. With a vaginoscopic entry approach the cervix, cervical canal, uterine cavity, endometrium, and both ostia were thoroughly inspected. Any abnormality was noted in reference to all of the above.

**Figure 3:**
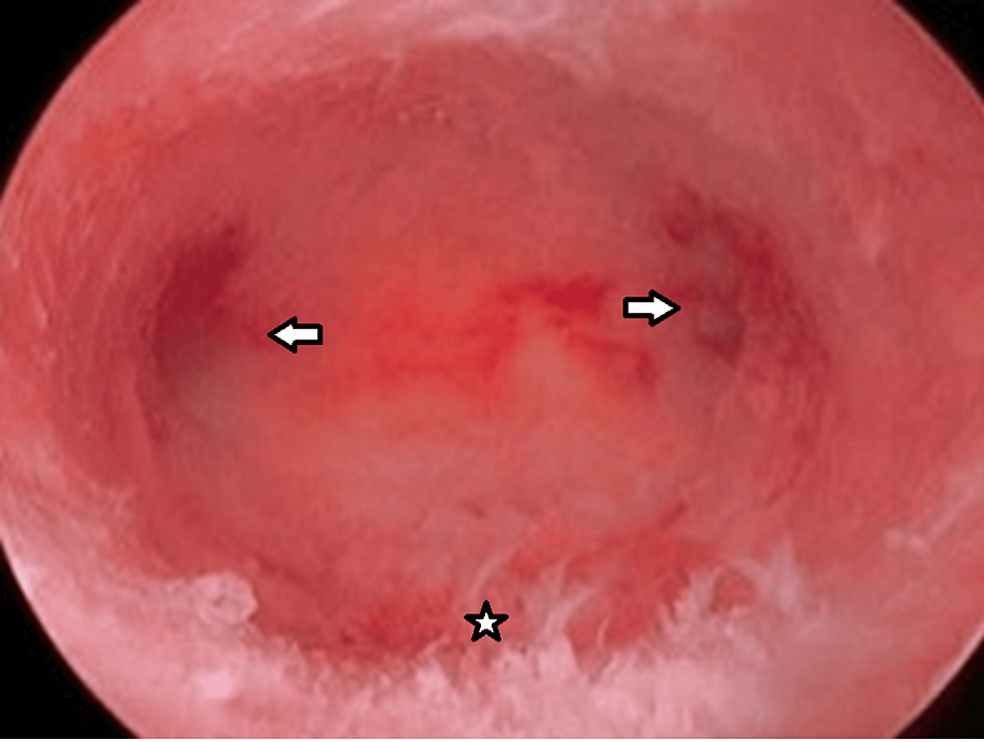
Normal hysteroscopic view Arrows indicating bilateral ostia and a star showing the normal endometrium.

Normal hysteroscopy was labeled where no pathology was seen and bilateral ostia were healthy-looking (Figure 3).

**Figure 4:**
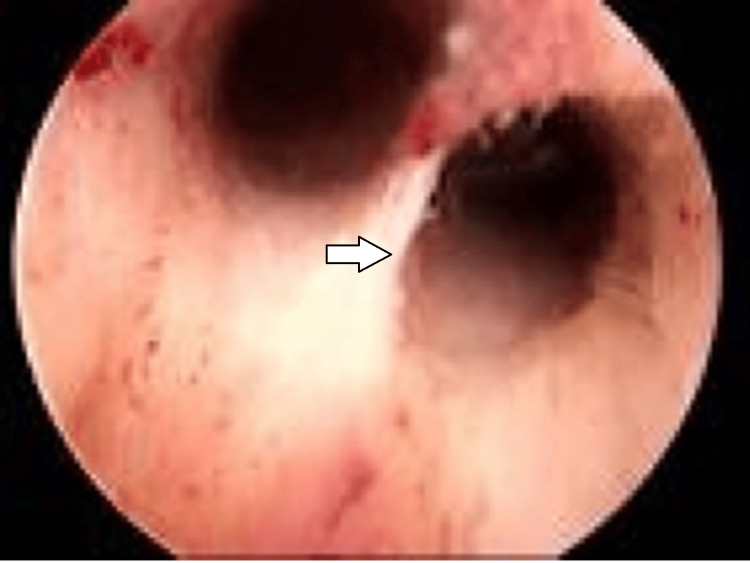
Abnormal hysteroscopy The arrow indicating at uterine septum.

Abnormal hysteroscopy was labeled wherever polyps or endometrial hyperplasia, septum, synechiae (intrauterine adhesion), and fibrosed ostia were observed (Figure 4).

Hysteroscopic findings were compared with laparoscopic findings for uterine and tubal pathology. Hysteroscopy as a procedure was also compared with laparoscopy as a one-step procedure for diagnostic accuracy in investigating a case of female infertility. The data was analyzed by Statistical Package for the Social Sciences (IBM SPSS Statistics for Windows, IBM Corp., Version 29.0, Armonk, NY). The association between hysteroscopy and laparoscopy was found by using the Chi-square test. The continuous variables were expressed as mean ± SD and categorical variables as proportions. The student’s t-test was used for the comparison of continuous variables and the Chi-square test for proportions.

## Results

In our study, out of a total of 75 cases evaluated for infertility, primary infertility (PI) patients were 48 (64%) and secondary infertility (SI) patients were 27 (36%). Age-wise distribution of the cases is shown in Table 2.

**Table 2:** Distribution of patients by age

Age of patients (years)	No. of patients of primary infertility (PI) (n=48)		No. of patients of secondary infertility (SI) (n=27)		Total (n=75)	
	No	%	No	%	No	%
18-25	14	18.66%	3	4%	17	22.66%
26-30	16	21.33%	11	14.66%	27	36%
31-35	16	21.33%	9	12%	25	33.33%
35-40	2	2.66%	4	5.33%	6	8%
Total	48	64%	27	36%	75	100%

In our study, the most common age group was between 26 and 30 years, and amongst them, PI and SI cases were 21.33% and 14.66% respectively. The least was seen in the age group of 36 to 40 years, where PI and SI cases were 2.6% and 5.33%.

A few general characteristics of the patients are shown in Table 3.

**Table 3:** General characteristics of the cases

Characteristics	Years
Mean age (years)	29.22 ± 4.7 years
Primary infertility	27.7 ± 4.5 years
Secondary infertility	30.7 ± 3.7 years
Mean duration of infertility (years)	5.06 ± 2.4 years
Primary infertility (years)	4.25 ± 2.9 years
Secondary infertility (years)	6.51 ± 3.2 years

In our study, the mean age of patients was 29.22 ±4.47 years. Patients in the SI group were slightly older compared to the primary group (30.7 ± 3.7 v/s 27.7 ± 4.5 years, P < 0.00013).

In the present study, the menstrual pattern showed that 36 (48%) cases had regular cycles followed by 27 (36%) had oligomenorrhea, 11 (14.66%) had menorrhagia and 1 (1.33%) had polymenorrhea.

In our study, out of 27 cases of SI, 15/27 (55.55%) had a previous cesarean delivery, 4/27 (14.81%) had a previous vaginal delivery, and 8/27 (29.63%) had previous miscarriages.

Table 4 lists the uterine findings on hysteroscopy.

**Table 4:** Uterine findings on hysteroscopy * Findings occurred alone or in combination

Uterine findings	No. of patients of primary infertility (PI) (n=48)		No. of patients of secondary infertility (SI) (n=27)	
Normal	32	42.66%	13	17.33%
Abnormal*	16	21.33%	14	18.66%
Ostia (fibrosed/blocked)	6	8%	5	6.66%
Intrauterine adhesions	5	6.66%	2	2.66%
Polyp	2	2.66%	2	2.66%
Myoma	0	0	2	2.66%
Endometrial (atrophic)	0	0	2	2.66%
Septum	2	2.66%	0	0
Small uterus	1	1.33%	0	0

Table 5 shows the findings on CPT.

**Table 5:** Tubal patency on laparoscopy chromopertubation (CPT) * fs - free spill, ds - delayed spill, rfs + rds - right side, lfs + lds - left side; CPT - chromopertubation

Tubal findings	Primary infertility (n=48)		Secondary infertility (n=27)	
Bilateral tubes				
Patent (fs+ds)*	17+22	49.33%	11+5	21.33%
Blocked	7	9.33%	6	8%
Unilateral tube patent				
Right (rfs+rds)*	1+0	1.33%	1+1	2.66%
Left (lfs+lds)*	2+1	4%	1+2	4%

In our study, both tubes were patent on CPT in PI vs SI in 49.33% vs 21.33% of total cases. Both tubes were blocked in PI vs SI in 9.33% vs 8% of total cases. The unilateral patent tube was present in PI vs SI in 5.33% vs 6.66% of total cases.

Laparoscopic findings in PI and SI are listed in Table 6. In our study, laparoscopy was normal in 13.33% of PI and 6.66 % of SI patients. The most common finding on laparoscopy was polycystic ovary syndrome (PCOD) (PI vs SI, 13.33% vs 9.33%). Bilateral cornual block was seen in PI vs SI (9.33% vs 4%). Unilateral cornual block was seen in PI vs SI (5.33% vs 4%).

**Table 6:** Laparoscopic findings

*Findings occurred alone or in combination (n = 75)	No. of patients of primary infertility (n=48)	No. of patients of secondary infertility (n=27)
Normal	10(13.33%)	5(6.66%)
Polycystic ovarian disorder (bulky ovaries)	10(13.33%)	7(9.33%)
Bilateral cornual block	7(9.33%)	3(4%)
Unilateral cornual block	4(5.33%)	3(4%)
Pelvic adhesions	3(4%)	2(2.66%)
Endometriosis	6(8%)	2(2.66%)
Uterine anomaly	1(1.33%)	1(1.33%)
Fitz-Hugh-Curtis syndrome	1(1.33%)	-
Myomas	2(2.66%)	1(1.33%)
Tubal abnormalities	4(5.33%)	3(4%)
	48	27

In our study, 20 patients (26.66%) underwent hysteroscopic intervention shown in Table 7. Adhesiolysis was the commonest procedure required in seven (9.33%) followed by hysteroscopic cannulation in six (8%).

**Table 7:** Hysteroscopic interventions

Interventions	No. of patients
Adhesiolysis	7
Polypectomy	3
Adhesiolysis + polypectomy	1
Hysteroscopic cannulation	6
Adhesiolysis + hysteroscopic cannulation	1
Septum resection	2
Total	20

In our study, a total of 30 procedures were performed in 20 patients during laparoscopy as listed in Table 8. The most common procedure required was ovarian drilling in 22.66% (17/75) followed by surgery for endometriosis in 10.66% (8/75). These procedures were based on the laparoscopic clinical findings. Adhesiolysis was required in 5/75 (6.66%).

**Table 8:** Laparoscopic interventions in our study (n = 20) * More than one procedure performed per patient

Interventions	Number*	% out of 75 interventions
Adhesiolysis	5	6.66
Surgery for endometriosis	8	10.66
Ovarian drilling	17	22.66

In our study, out of 75 participants, 44 were found to be normal for both hysteroscopy and laparoscopy, and two were abnormal in both interventions. Additionally, 28 were abnormal for hysteroscopy but found normal in laparoscopy, while one was normal for hysteroscopy but abnormal for laparoscopy, as shown in Table 9. During the statistical analysis, the Chi-square test value was calculated as 0.925, which is higher than the P-value of 0.05. The test result indicates a non-significant statistical correlation. Therefore, this study is unable to establish a statistical correlation between the two interventions.

**Table 9:** Comparison of uterine findings of laparoscopy versus hysteroscopy

Intervention		Hysteroscopy		Total
		Normal	Abnormal	
Laparoscopy	Normal	44	28	72
	Abnormal	1	2	3
Total		45	30	75

## Discussion

The evaluation and treatment of infertility has changed dramatically over the past few decades, due to a greater number of women attempting pregnancy at older ages, with the introduction of newer modalities like in vitro fertilization (IVF) and other assisted reproductive techniques (ARTs). Despite recent achievements in technology for the evaluation of infertile women, no single intervention could completely explore all parts of the female genital tract. Each has its limitations, risks, and fallacies; hence, the quest for an optimized diagnostic tool that can cause the least inconvenience with maximum benefits to women is the need of the hour.

Hence, our study focused on a detailed hysterolaparoscopic survey of 75 women of PI and SI. In our study group (n=75), the incidence of PI vs SI was (64% vs 36%). A similar study by van Kessel et al. [9] (n=300) observed that 69% had PI while 31% had SI.

In our study group (n=75), the mean age was 29.22 ± 4.47 years, the mean age of the PI group was 27.7 ± 4.5 years, and the mean age of the SI group was 30.7 ±3.7 years. Various studies by other authors namely, Kabadi et al. [10] reported a mean age of 26.8 years; while in a study by Puri et al. [11] mean age was 28.5 years.

Hysteroscopic findings

Diagnostic hysteroscopy offers a reliable evaluation of the uterine cavity and subsequent detection of intrauterine disease [12]. Complications rates of diagnostic hysteroscopy are as low as 0.012% [13]. The mean prevalence of uterine malformation in the general population and the population of fertile women is approximately 3.5%, in infertile patients approximately 5.2% and in patients with recurrent pregnancy losses approximately 13% [14].

In our study (n=75), hysteroscopic findings showed that 60% had normal findings, out of which 42.66% were of PI and 17.33% were of SI.

Anomalies of the uterus are considered to be one of the reasons for infertility in women, and for this reason, diagnostic hysteroscopy is fundamental in screening for infertility [15]. With the view of the low complication rates, minimal time requirement, and negligible effect on the post-operative course, hysteroscopy can be performed on all infertile patients undergoing diagnostic laparoscopy. Table 10 shows the comparative analysis of hysteroscopic findings with previously published studies.

**Table 10:** Table showing comparative study of hysteroscopic findings by various authors

Hysteroscopic findings	Present study (n=75)	Dawle et al. [16]	Firmal et al. [17]	Ugboaja et al. [18]	Ekine et al. [19]
Endometrial polyp	4(5.33%)	26(7%)	6(9%)	3(6%)	8(11.4%)
Submucous fibroid	2(2.66%)	-	1(1%)	1(2%)	3(4.2%)
Septate uterus	2(2.66%)	7(1.8%)	2(3.2%)	2(4%)	1(1.4%)
Tubal ostial block	13(14.66%)	-	-	-	1(1.4%)
Intrauterine adhesions	7(9.33%)	-	-	-	-
Normal	45(60%)	-	-	44(88%)	55(79%)

However, hysteroscopy does not diagnose all pathologies likely to contribute to infertility.

Its conjoint association with other investigations may lead to a better focus on the diagnosis of infertility. Hence the value of hysteroscopy undoubtedly scores higher than HSG as it can pick up the lesions that are likely to be missed by HSG. The advantage of hysteroscopy is that intrauterine adhesions can be dealt with therapeutically in the same setting.

The abnormal findings that were detected on hysteroscopy were dealt with therapeutically at the same sitting in 26.66% (20/75) patients which include adhesiolysis, septum resection, and polypectomies. This was a significant advantage of hysteroscopy over laparoscopy.

Laparoscopy findings

A comparative analysis was illustrated in Table 11 by similar studies in the past based on findings on laparoscopy.

**Table 11:** Table showing comparative study of laparoscopy findings by various authors

Laparoscopy findings	Present study	Ngowa et al. [20]	Ludwin et al. [21]	Kavitha et al. [22]
Polycystic ovarian disorder	16(21.33%)	-	12(19%)	24(34.2%)
Endometriosis	8(10.66%)	51(14%)	5(8%)	8(11.4%)
Bilateral tubal block	13(17.33%)	18(5%)	10(16%)	8(11.4%)
Unilateral tubal block	7(9.33%)	30(8%)	12(19.3%)	4(5.7%)
Pelvic adhesions	5(6.66%)	40(11%)	7(11.2%)	5(7.1%)
Myoma	3(4%)	31(8%)	4(6.45%)	4(5.7%)
Uterine anomaly	1(1.33%)	19(5%)	8(12.9%)	2(2.8%)
Fitz-Hugh-Curtis syndrome	1(1.33%)	23(6%)	-	1(1.4%)
Normal	10(13.33%)	-	-	14(20%)

Findings that may have had implications as a cause of infertility like pelvic adhesions, fimbrial agglutination, endometriosis, PCOD, and hydrosalpinx were detected in a significant number of patients and were dealt with surgically in the same sitting. The interventions for these additional findings were adhesiolysis, surgery for endometriosis, and ovarian surgeries like drilling, and ovarian cystectomies (Table 7).

The Chi-square test was used for statistical analysis, and the calculated test value is 0.925, which is greater than the chosen significance level of 0.05. In this case, the non-significant result (p > 0.05) suggests that there is not enough evidence to establish a statistical correlation between the two interventions. In other words, the study did not find a significant difference between hysteroscopy and laparoscopy. That has proved that these two interventions are not mutually exclusive, but rather complementary to each other.

The results of our study bring us to the conclusion that on the whole, both hysteroscopy and laparoscopy are important adjunctive methods in the diagnosis of infertility with the added advantage of the therapeutic potential.

Diagnostic hysterolaparoscopy is a safe and effective tool for the comprehensive evaluation of infertility. it is a straightforward way to identify the correctable organic pathologies. As it can help in detecting peritoneal endometriosis, adnexal adhesions, and septum in the uterus. These correctable abnormalities can unfortunately easily be missed by routine pelvic examinations and usual imaging procedures. So, this intervention tool can detect various structural abnormalities in multiple sites like the pelvis, tubes, and the uterus as a one-step procedure. Hystero-laparoscopy can be considered a definitive investigative daycare procedure for the evaluation of female infertility, when done by experienced hands and with a proper selection of patients.

Limitations

This study did not include a direct comparison between hysteroscopy and laparoscopy as two separate interventional entities. Laparoscopy is also an invasive modality requiring general anesthesia.

## Conclusions

The results of our study bring us to the conclusion that hysterolaparoscopy as a "one-step" procedure is recommended as the primary procedure in evaluating infertile women. Laparoscopy is a better investigative procedure for a panoramic view of gross pelvic anatomy, particularly tubal pathology, uterus, and peritoneal cavity. It has the advantage of not only detecting additional factors but also providing therapeutic benefits in the same setting. The addition of hysteroscopy to laparoscopy is invaluable in the infertility workup as it has a definite edge in the detection of intrauterine pathology, as well as being therapeutic at the same time. More accuracy in the diagnostic findings and therapeutic intervention in the same setting will help in reducing the time and cost of treatment.
